# Shared Core Microbiome and Functionality of Key Taxa Suppressive to Banana Fusarium Wilt

**DOI:** 10.34133/2022/9818073

**Published:** 2022-09-15

**Authors:** Zongzhuan Shen, Linda S. Thomashow, Yannan Ou, Chengyuan Tao, Jiabao Wang, Wu Xiong, Hongjun Liu, Rong Li, Qirong Shen, George A. Kowalchuk

**Affiliations:** ^1^Jiangsu Provincial Key Lab of Solid Organic Waste Utilization, Jiangsu Collaborative Innovation Center of Solid Organic Wastes, Educational Ministry Engineering Center of Resource-Saving Fertilizers, The Key Laboratory of Plant Immunity, Joint International Research Laboratory of Soil Health, Nanjing Agricultural University, Nanjing, 210095 Jiangsu, China; ^2^The Sanya Institute of the Nanjing Agricultural University, Sanya, Hainan Province, China; ^3^U.S. Department of Agriculture, Agricultural Research Service, Wheat Health, Genetics and Quality Research Unit, Pullman, WA, USA; ^4^Ecology and Biodiversity Group, Institute of Environmental Biology, Department of Biology, Utrecht University, 3584 CH Utrecht, Netherlands

## Abstract

Microbial contributions to natural soil suppressiveness have been reported for a range of plant pathogens and cropping systems. To disentangle the mechanisms underlying suppression of banana Panama disease caused by *Fusarium oxysporum* f. sp. *cubense* tropical race 4 (Foc4), we used amplicon sequencing to analyze the composition of the soil microbiome from six separate locations, each comprised of paired orchards, one potentially suppressive and one conducive to the disease. Functional potentials of the microbiomes from one site were further examined by shotgun metagenomic sequencing after soil suppressiveness was confirmed by greenhouse experiments. Potential key antagonists involved in disease suppression were also isolated, and their activities were validated by a combination of microcosm and pot experiments. We found that potentially suppressive soils shared a common core community with relatively low levels of *F. oxysporum* and relatively high proportions of Myxococcales, Pseudomonadales, and Xanthomonadales, with five genera, *Anaeromyxobacter*, *Kofleria*, *Plesiocystis*, *Pseudomonas*, and *Rhodanobacter* being significantly enriched. Further, *Pseudomonas* was identified as a potential key taxon linked to pathogen suppression. Metagenomic analysis showed that, compared to the conducive soil, the microbiome in the disease suppressive soil displayed a significantly greater incidence of genes related to quorum sensing, biofilm formation, and synthesis of antimicrobial compounds potentially active against Foc4. We also recovered a higher frequency of antagonistic *Pseudomonas* isolates from disease suppressive experimental field sites, and their protective effects against banana *Fusarium* wilt disease were demonstrated under greenhouse conditions. Despite differences in location and soil conditions, separately located suppressive soils shared common characteristics, including enrichment of Myxococcales, Pseudomonadales, and Xanthomonadales, and enrichment of specific *Pseudomonas* populations with antagonistic activity against the pathogen. Moreover, changes in functional capacity toward an increase in quorum sensing, biofilm formation, and antimicrobial compound synthesizing involve in disease suppression.

## 1. Introduction

Soil-borne diseases can severely impact global crop production across a wide range of crops, cropping systems and disease-causing agent, and these impacts are expected to increase under conditions of climate change [1]. Plant diseases are estimated to be responsible for as many as twenty percent of global food production lost annually [2]. As one of the most devastating soil-borne pathogens across many agricultural production systems in the world, *Fusarium* spp. could attack a range of important crops resulting in damping-off, root rot, and vascular wilt [3]. Banana *Fusarium* wilt, also known as Panama disease, was caused by the infection of *Fusarium oxysporum* f. sp. cubense tropical race 4 (Foc4) [4]. This *Fusarium* wilt is notably hard to control, probably due to the pathogen which could produce chlamydospores that can survive in soil for decades [5]. Plant root-associated microbiomes are increasingly recognized as a possible dominator of natural pathogen suppression and have become a goal for innovative ways aimed at improving disease management [6]. Despite evidence that many biocontrol agents can contribute to relieving the damage caused by *Fusarium* wilt [7], a main problem is that their efficacy is unstable under field conditions. Therefore, it is important for us to improve our ability in predicting and engineering microbiome functions to increase soil suppressiveness.

Disease-suppressive soil is the best evidence that illustrating microorganisms participated in the plant protection against soil-borne pathogens. Disease-suppressive soil has been defined as those in which disease incidence or severity still maintains at a low level, even in the presence of the pathogen, susceptible host crop, and climatic condition conducive to disease occurrence [8]. With the understanding of the necessity to reduce the application of chemical pesticides, suppressive soil has emerged as a research hotspot with important implications in the development of more sustainable agriculture [9]. Disease-suppressive soil could provide a basis for manipulating soil community to generate sustainable alternate strategies for pathogen control, and soils suppressive to soil-borne pathogens have been discovered for a range of crops across many locations [10–12]. Although banana-suppressive soils to *Fusarium* wilt have previously been reported [13–15], it is still challenging to unravel the complicated microbial mechanisms with regard to disease suppression.

With the development of DNA sequencing techniques, it has become more feasible to describe the microbial consortia potentially responsible for disease suppression across a range of pathosystems [16]. The rhizosphere represents the zone of interaction between the soil microorganisms and plant roots, and it has therefore been the focus of studies relating soil-borne communities to disease suppressive capacities [9, 10, 17]. Such studies have typically examined specific natural disease-suppressive soils, attributing disease-suppressive properties to the presence of specific antagonistic microorganisms, such as *Burkholderia* [18], *Lysobacter* [17], and *Streptomyces* [9]. However, soil communities are known to vary greatly across sites determined by a range of abiotic and biotic factors [19]. It is generally unknown whether there are common features of disease-suppressive soils against target pathogens across agricultural fields that differ in location and edaphic factors.

**Figure 1:**
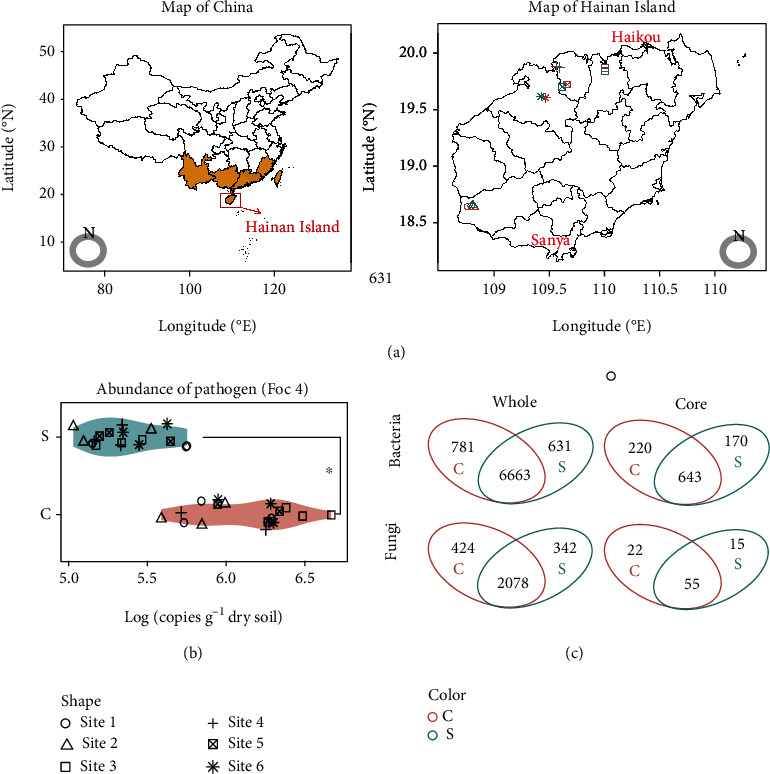
Distribution of soil sampling sites and overview of the composition of the core bacterial and fungal communities. (a) Map showing the location of selected orchards. Yellow areas represent the main banana production regions in China. (b) Violin plot depicting the mean abundance of Foc4 in disease-suppressive (*S*) and -conducive (*C*) soils. The ^∗^ indicates a significant difference between *C* and *S* orchards according to Wilcoxon tests. (c) Venn diagram exhibiting the unique and shared bacterial and fungal OTUs between conducive and suppressive orchards for the total and core microbiomes. *C*_whole_ and *S*_whole_ represent all identified OTUs within the microbiome, while *C*_core_ and *S*_core_ represent OTUs designated as being part of the core microbiome.

To expand our understanding about the general microbial properties of disease-suppressive soils, we aimed to (1) investigate whether separately located disease-suppressive agricultural soils shared common microbial community features, (2) identify keystone microbial groups potentially involved in disease suppression across different field sites, and (3) verify the ability of identified keystone microbes to suppress *Fusarium* wilt disease. With these goals in mind, the microbiome across six agricultural sites under long-term monocropping of banana in Hainan Island, China, was examined (Figure 1(a)). Each site included paired orchards, one potentially suppressive to banana wilt and the other conducive to the disease. We used both community-based sequencing and classical approaches to decipher the geographically distributed *Fusarium* wilt-suppressive soils. Common features of disease-suppressive soil were first used to identify microbial taxa potentially involved in disease suppression, and subsequent cultivation-based microcosm experiments were used to assess the potential of some of these taxa to suppress the pathogen. In total, we sought to reveal how core features of the soil microbiome may impact pathogen suppression across a range of field conditions and sites.

## 2. Results

### 2.1. Soil Properties and Microbial Biomass

Potentially disease-suppressive soils from the six geographically separated banana orchards differed in their soil properties and edaphic factors (Table S1). However, in comparison to the disease-conducive soils, disease-suppressive soils exhibited a higher pH and higher contents of available phosphorus (AP), available potassium (AK), total carbon (TOC), and total nitrogen (TON) on average. Although the differences in bacterial and fungal abundances were not significant, disease-suppressive soils together showed significantly higher ratios of bacteria to fungi (*B*/*F*) compared to disease-conducive soils based on quantitative PCR assays (Figure S1). Furthermore, disease-suppressive soils together harbored a significantly lower abundance of Foc4 compared to disease-conducive soils (Figure 1(b)).

### 2.2. Shared Core Microbiome Features across Disease-Suppressive Soils

Amplicon sequencing resulted in 8,075 bacterial and 2,844 fungal OTUs in total across all disease-suppressive and -conducive soils based on 97% similarity after basic quality control (Figure 1(c)). The rarefaction curves for each sample nearly approached saturation, indicating that the sequencing data were reasonable for evaluating the microbial diversity and composition (Figure S2). Given many rare taxa only detected in specific locations, we further sought to examine features of the core microbiome across all sites. OTUs that appear as in at least 80% of all soil samples were defined as belonging to the core microbiome. A total of 1,033 bacterial OTUs sequences and 92 fungal OTUs were deemed to constitute the core microbiomes for pooled disease suppressive and conducive soils (Figure 1(c), Table S2). No significant difference was observed for Shannon index of core bacterial and fungal communities (Figure S3). The core bacterial microbiome consists of Acidobacteria, Actinobacteria, Bacteroidetes, Firmicutes, Gemmatimonadetes, Proteobacteria, and other low relative abundance phyla while the core fungal microbiome was mainly comprised by Ascomycota, Basidiomycota, Glomeromycota, and Zygomycota both in the potentially conducive and suppressive soils (Figure S4).

**Figure 2:**
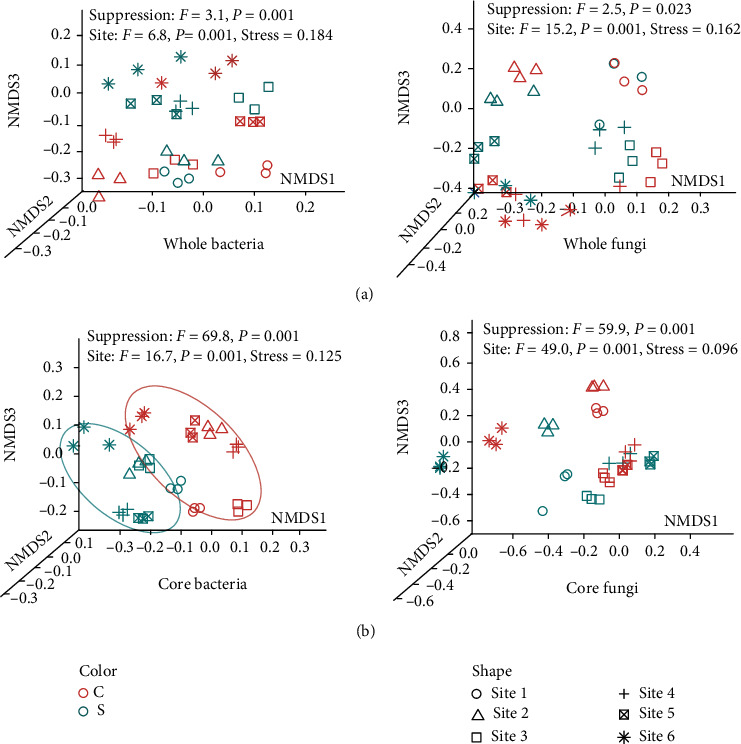
Structure of whole and core microbiome in disease-suppressive and -conducive soils. (a) Nonmetric multidimensional scaling (NMDS) ordination plots displaying the composition differences for whole bacterial and fungal community calculated using weighted UniFrac distances. (b) NMDS plot displaying the composition differences for core bacterial and fungal community calculated using weighted UniFrac distances.

Nonmetric multidimensional scaling ordination (NMDS) analysis exhibited distinct differences in the composition of bacterial and fungal community with respect to site location site and disease-suppressive ability. Using the full bacterial and fungal community datasets, field location was found to be the first factor distinguishing microbial community structure (Figure 2(a), Table S3). However, when only considering the core communities, core bacterial communities from disease-suppressive soils were grouped together, with a clear separation from those of the disease-conducive soils (Figure 2(b), Table S3). Despite both location and suppressive ability were significant drivers for total and core bacterial and fungal communities, however, location was more important for total communities while suppressive ability was more so for the core communities, especially for bacterial communities.

### 2.3. Potential Key Species within Shared Core Microbiomes of Disease-Suppressive Soils

**Figure 3:**
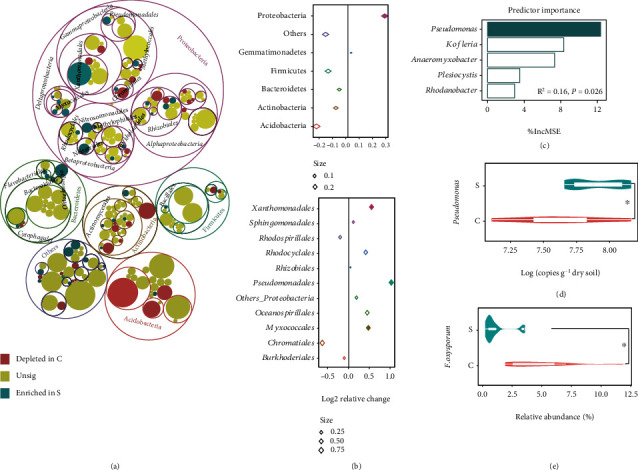
Key taxa for the core microbiome in disease-suppressive soils. (a) Taxonomic differences for the core bacterial microbiome between disease-suppressive and -conducive soils are based on 16S rRNA gene sequences. The outside circles to inner circles represent phylum, class, order, and family level, respectively. Solid circles represent genera. (b) Bubble graph showing the log2 transformed relative changes of relative abundance of dominate phyla and orders in significantly enriched phylum of Proteobacteria in disease suppressive to conducive soils for core bacterial community. Rhombus size indicates absolute value of the log2 transformed relative changes. Solid rhombus indicates that the taxa were significantly enriched in disease-suppressive soils. (c) Random forest mean predictor importance of relative abundance of significantly enriched genera among the Myxococcales, Pseudomonadales, and Xanthomonadales as drivers for the abundance of Foc4 in soil. Solid bar represents that the specific predictor is significant. MSE: mean square error. (d) Violin plot showing the abundance of *Pseudomonas* in disease-suppressive (*S*) and -conducive (*C*) soils. The ^∗^ indicates a significant difference between *C* and *S* treatments based on Wilcoxon tests. (e) Violin plot showing the relative abundance of *F. oxysporum* in the core microbiome in disease-suppressive and -conducive soils. The ^∗^ indicates a significant difference between *C* and *S* treatments based on Wilcoxon tests.

Among the dominant phyla of core bacterial communities, a significantly higher relative abundance of Proteobacteria and lower relative abundance of Acidobacteria on average were observed in potentially suppressive soils (Figure 3(a), Table S4). Further, the results of relative change analysis displayed that Proteobacteria were only significantly enriched in disease-suppressive soils (Figure 3(b)). Within the Proteobacteria, the Myxococcales, Pseudomonadales, and Xanthomonadales showed significantly higher relative abundance in suppressive soils as compared to conducive soils when examining relative changes at the order level (Figure 3(b), Table S5). Within these three enriched orders, five genera, *Anaeromyxobacter*, *Kofleria*, *Plesiocystis*, *Pseudomonas*, and *Rhodanobacter*, were found to be significantly enriched in disease-suppressive soils as compared to conducive soils (Figure S5). Further, random forest analysis showed that the enrichment of *Pseudomonas* in potentially disease-suppressive soils was the most important variable for predicting the reduced abundance of Foc4 among these five genera (Figure 3(c)). Moreover, quantitative PCR results showed that potentially disease-suppressive soils contained higher abundances of *Pseudomonas* than disease-conducive soils (Figure 3(d)). In addition, potentially suppressive soils harbored a significantly lower relative abundance of *F. oxysporum* on average (Figure 3(e)).

### 2.4. Transfer of Disease Suppressiveness

**Figure 4:**
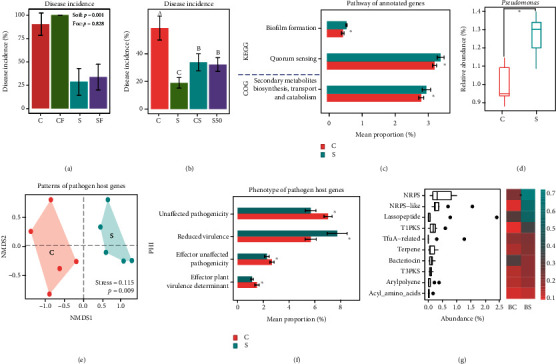
Functionalities in disease-suppressive and -conducive soils. (a) Histogram showing the percentage (mean ± standard error of four replicates) of banana tissue seedlings with *Fusarium* wilt symptoms in conducive soil (*C*), conducive soil inoculated with Foc4 (CF), suppressive soil (*S*), and suppressive soil inoculated with Foc4 (SF). (b) Histogram showing the percentage of banana tissue seedlings with *Fusarium* wilt symptoms in conducive soil (*C*), suppressive soil (*S*), conducive soil mixed with 10% (w/w) suppressive soil (CS), or suppressive soil heated to 50°C (S50). Different letters above the bars mean significant differences (*p* < 0.05, ANOVA test). (c) Pathways that are significantly enriched in banana orchards suppressive to *Fusarium* wilt when metagenome sequences were blasted against KEGG and COG databases. The ^∗^ above the bar represents a significant difference between *C* and *S* treatments based on ANOVA test. (d) Boxplot showing the relative abundance of *Pseudomonas* in soils suppressive or conducive to *Fusarium* wilt as final metagenomic dataset blasted against the NR database. (e) NMDS ordination plot displaying the functional differences between suppressive and conducive soils based on metagenomic analysis of genes related to the *Pseudomonas* community when blasted against the PHI database. (f) Bar plot showing the phenotypic differences between suppressive and conducive soils according to metagenomic analysis of genes related to the *Pseudomonas* community when blasted against the PHI database. (g) The boxplot in the left panel displaying the top 10 abundant secondary metabolites in the soils on average based on the antiSMASH database. The heat map in the right panel showing the top 10 abundant secondary metabolites related to antimicrobial compounds synthesizing in the disease conducive (*C*) and suppressive (*S*) soils. The heat map was plotted according to the mean intensity, and the ^∗^ in the heat map cell symbol indicates a significant difference between C and S treatments.

A low level of disease incidence was observed when banana plants were grown in disease-suppressive soil even inoculated with Foc4 under greenhouse conditions. In contrast, nearly all plants became diseased when conducive soils were amended with a Foc4 spore suspension (Figure 4(a)). Further, disease suppressiveness of the disease-suppressive soil was partially lost when the soil was heated to 50°C. In soil-transfer experiments, in which 10% of suppressive soil was mixed with conducive soil before plant cultivation, disease suppressiveness was partially transferred (Figure 4(b)). Collectively, these results support the notion that observed disease suppressiveness toward Foc4 is biological in nature.

### 2.5. Functional Traits of the Soil Microbiome in Disease-Suppressive Soil

Shotgun sequencing of the disease-suppressive and -conducive soils from the experimental field site 3 resulted in an average of 17.2 GB paired-end reads per sample, totaling 172 GB high-quality reads after quality control (Table S6). The filtered sequences were de novo assembled to yield an average of 114,849 contigs, and an average of 176,417 ORFs was generated for each sample (Table S7). When the final sequences were blasted against the COG database, a higher fraction of pathways related to the biosynthesis of secondary metabolites was found in the suppressive soil as compared to conducive soil. When blasting DNA reads against the KEGG database, a higher frequency of pathways involved in quorum sensing and biofilm formation was observed in the disease-suppressive soil (Figure 4(c)). Taxonomic assignment of the annotated DNA reads also identified members of *Pseudomonas* as more abundant in the disease-suppressive soil as compared to the conducive soil (Figure 4(d)). NMDS analysis was also performed on functional genes related to *Pseudomonas* as determined using the PHI database, revealing a clear distinction in *Pseudomonas* functional traits that was observed between disease-suppressive and -conducive soils (Figure 4(e)). Interestingly, genes involved in “unaffected pathogenicity” and “effector of plant virulence determinants” were significantly depleted in the suppressive soil, and genes related to reduced virulence were significantly enriched in these samples (Figure 4(f)). A further secondary metabolite analysis by using antiSMASH exhibited that NRPS was significantly enriched in the disease-suppressive soil compared to disease-conducive soil (Figure 4(g)).

### 2.6. Pseudomonas Isolates from Disease-Suppressive Soils and Their Abilities to Suppress Foc4 Invasion

**Figure 5:**
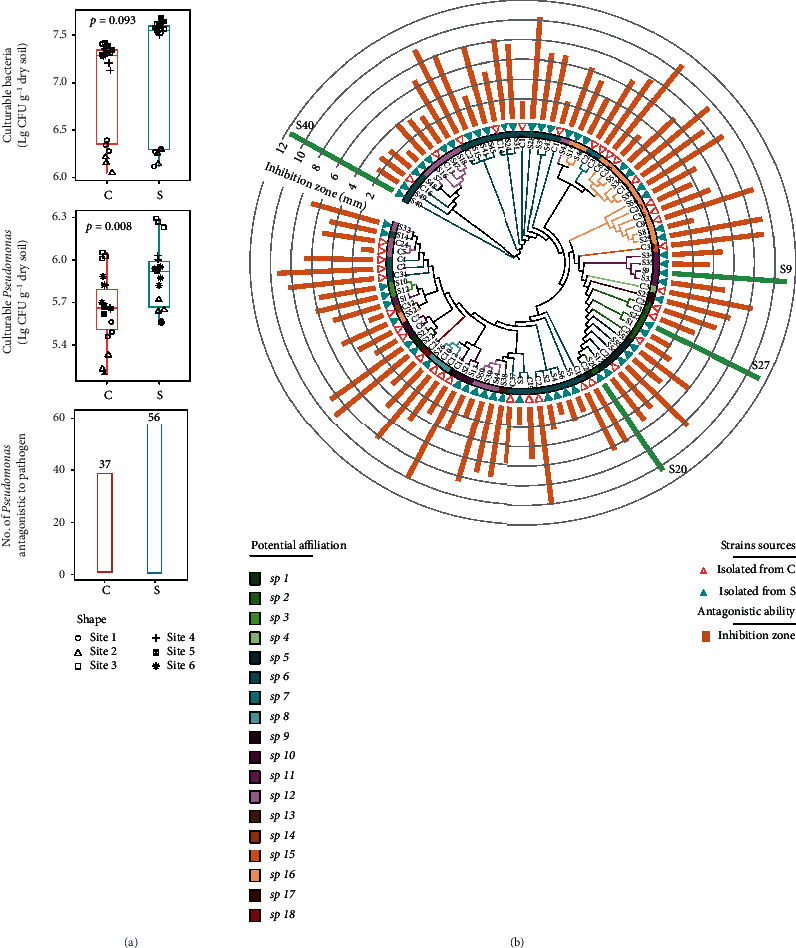
Isolation and identification of antagonistic *Pseudomonas* spp. in disease-suppressive and -conducive soils. (a) Histogram exhibiting the culturable counts of bacteria and *Pseudomonas* in conducive (*C*) and suppressive (*S*) soil, and the number of antagonistic *Pseudomonas* antagonistic to Foc4 isolated from *C* and *S* field soils. (b) Cladogram depicting potential phylogenetic relationships between isolated *Pseudomonas* strains in disease-suppressive or -conducive soils. Leaf labels are representative IDs of each strain. The inner rings indicate the potential species-level taxonomy according to 16S rRNA genes, and the outer ring represents the soil from which the strain was isolated. The bar plot above each node represents the antagonistic ability against Foc4. The length of each bar was plotted based on the inhibition zone of the *Pseudomonas* strain against Foc4. Solid and empty triangles represent the strains isolated from *C* or *S* treatments, respectively.

Although the counts of culturable bacteria between disease-conducive and -suppressive soils were not significantly differed, disease-suppressive soils exhibited higher counts of culturable *Pseudomonas* compared to conducive soils (Figure 5(a)). Results of dual culture assays revealed that only 6.4% of isolated *Pseudomonas* strains were antagonistic to the Foc4, with 56 antagonistic strains recovered from disease suppressive soils and 37 strains from conducive soils (Figure 5(a)). Subsequently, four antagonistic *Pseudomonas* strains S9, S20, S27, and S40 from disease-suppressive soils were selected for further analysis based upon the fact that they exhibited the largest inhibition zones (>10 mm) (Figure 5(b)).

**Figure 6:**
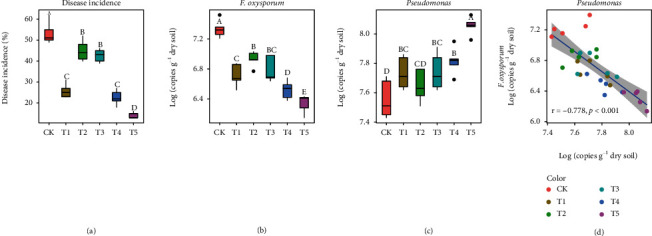
Effects of selected antagonistic *Pseudomonas* spp. on disease suppression. (a) Boxplot showing the disease incidence of different treatments amended with different antagonistic *Pseudomonas* isolates. Different letters above the boxes indicate statistically significant differences (ANOVA, *p* < 0.05). Treatments of T1, T2, T3, and T4 represent monocropped soil amended with 50 mL fermentation liquor of the *Pseudomonas* strains S9, S20, S27, and S40, respectively. T5 represents monocropped soil amended with 50 mL equally mixed fermentation liquors from all above four strains (T5). CK represents control of monocropped soil amended with 50 mL sterile KB broth. (b) Boxplot showing the abundance of *F. oxysporum* in the rhizosphere soils from different treatments. (c) Boxplot showing the abundance of *Pseudomonas* in the rhizosphere soils from different treatments. (d) Scatter diagram depicting the correlations between the relative abundance of *Pseudomonas* and the abundance of *F. oxysporum* in the rhizosphere soils.

A greenhouse experiment showed that the application of each of these four antagonistic *Pseudomonas* strains to field soil could significantly decreased the incidence of banana *Fusarium* wilt three months after seedlings were transplanted. Compared to controls without introduced *Pseudomonas* strains (CK), treatment (T5) with the mixture of fermentation cultures from these four antagonistic *Pseudomonas* strains displayed the lowest disease incidence, with a 39.4% decrease of disease (Figure 6(a)). Quantitative PCR results showed that all treatments amended with antagonistic *Pseudomonas* strains displayed reduced abundance of *F. oxysproum* in the banana rhizosphere soil compared to the CK treatment (Figure 6(b)). Also, treatments T1, T3, T4, and T5 exhibited a greater abundance of *Pseudomonas* in the rhizosphere compared to the CK treatment (Figure 6(c)). These results resulted in a significantly negative correlation between the abundance of *Pseudomonas* and *F. oxysporum* in the banana rhizosphere soil of this experiment (Figure 6(d)).

## 3. Discussion

The diversity of plant and microbiome in natural ecosystems can help maintain low levels of disease [20], but the intensive agriculture that continuously growing with a little number of crop varieties leads to an outbreak of soil-borne diseases caused by plant pathogens. With consecutive cropping of the same crop cultivar or application of soil amendments, monocropped soils can achieve disease suppression as a result of the selection and enrichment of antagonistic microorganisms or overall microbial diversity that can suppress the soil-borne pathogen [21]. Previous studies have often been confined to the examination of microbial composition in disease-suppressive soils of specific crops from a single site. However, different fields of the same cropping system can vary greatly in their soil characteristics and hence overall soil-borne microbial community composition [22, 23]. Thus, we still have a relatively limited understanding of whether signatures of suppressive soils are held in common across different field sites. The microbial compositions in *Fusarium* wilt-diseased and disease-free banana orchards have been explored previously [24, 25]; however, this is the first study that attempts to investigate whether geographically distributed agricultural soils suppressive to *Fusarium* wilt exhibit community features that can be connected with their disease-suppressive capacities.

In the current study, NMDS of the full bacterial and fungal communities across the different sites revealed that the location site was the dominating driver of microbial community patterns. This observation is in line with previous reports that soil characteristics and large-scale distribution patterns are the principal drivers of total community structure in soil [26, 27]. However, when considering the core microbiome, consisting of 1,033 bacterial OTUs and 92 fungal OTUs, we found disease-suppressive capacity to be a major determinant of the community structure. This finding is consistent with the notion that key characteristics of the core rhizosphere microbiome can develop independently of larger course-scale environmental drivers [28]. Core microbiomes were recently proposed as sets of microbial taxa that play similar functions at the ecosystem level, and they have been recognized across a range of various environmental factors [29]. The soils suppressive to banana *Fusarium* wilt disease across a range of locations shared feature of their core microbial community, suggesting that disease suppressive soils may involve the selection of a set of ubiquitous microbial species involved in disease suppression under the stress of pathogen invasion.

Similar to other studies using community-based analyses of core bacterial soil communities [30–32], the core bacteria taxa in our study were dominated by Acidobacteria, Actinobacteria, Bacteroidetes, Firmicutes, Gemmatimonadetes, and Proteobacteria. Although no other reports have been focused on core fungal communities, the core fungal community was mainly comprised by Ascomycota, Basidiomycota, Glomeromycota, and Zygomycota in our study. Our results suggest that the core microbiome of these wilt-suppressive soils contains fundamental taxonomic groups probably involved in supporting basic soil functions [33–35].

As previously found in studies of banana Panama disease [36], disease-suppressive soils had lower proportion of *F. oxysporum* as compared to the disease-conducive soils. Our results demonstrated that Proteobacteria in the core bacterial community are significantly enriched in banana soils suppressive to banana Panama disease, which is in agreement with previous studies suggesting that members of the gamma-proteobacteria can be considered as keystone species of healthy banana plants in *Fusarium* wilt-infested orchards [37, 38]. We further found that the relative abundances of Myxococcales, Pseudomonadales, and Xanthomonadales within the Proteobacteria were significantly increased in disease-suppressive soils. As many isolates from these three orders were considered as the most promising biocontrol agents [39, 40], our results suggest that members of Myxococcales, Pseudomonadales, and Xanthomonadales may be involved in maintaining the soil suppressiveness to banana *Fusarium* wilt.

The culture-independent approaches employed in this study, including random forest analysis based on amplicon sequencing results and taxonomic analysis of shotgun sequencing results, showed that *Pseudomonas* may play crucial roles in determining soil suppressiveness of in soils suppressive to banana Panama disease. Furthermore, culture-dependent analyses demonstrated that isolates of *Pseudomonas* from suppressive soils with the capacity to suppress the growth of Foc4 could protect banana from pathogen invasion. *Pseudomonas* populations have constantly been recognized to be involved in the suppression of *Fusarium* wilt disease or wheat take-all disease [41, 42], as well as playing a vital role against pathogen infection in the banana endophytic microbiome [43]. One possible mechanism behind wilt suppression may be the production of antimicrobial compounds [44]. In agreement with this, we found that the microbiome in disease-suppressive soils had a higher proportion of functional genes linked to the biosynthesis of secondary metabolites. Another possible mechanism could be the occupation of niches overlapping with the pathogen thereby leading to competition for instance for available resources [45]. It was reported that beneficial microbes may preferentially colonize the root niches and compete available resources via formatting biofilm to suppress the pathogen invasion [46]. In line with this, we observed that disease-suppressive soils displayed a higher frequency of functional genes relating to biofilm formation. Both biofilm formation and the production of many antibiotics by gram-negative bacteria are linked to quorum sensing [47, 48]. Interestingly, we also found higher frequencies of genes for quorum sensing in our suppressive soils, indicating that it likely plays a key role in suppressing pathogen invasion.

Notably, despite the high population size of *Pseudomonas* in disease suppressive as compared to conducive soils, only a small percentage of *Pseudomonas* isolates with antagonistic activity *in vitro* were recovered in the present study, in agreement with a previous report that not all *Pseudomonas* spp. isolates directly inhibit pathogens [49]. Although more antagonistic *Pseudomonas* isolates were found in the suppressive than in the conducive soils, the quorum sensing and biofilm of *Pseudomonas* probably contribute to the soil suppressiveness as well. Hence, we suggest that *Pseudomonas* spp. in the suppressive soil were stimulated under the pressure of the pathogen and by interactions with the indigenous microbiome. In agreement with this hypothesis, a clear difference in pathogenicity and virulence of *Pseudomonas* communities was observed in our study, generally supporting the idea that the function of the microbiome is redundant and probably can be directed in specific directions under biotic or abiotic stress [50].

Thus, by combining cultivation-dependent and independent approaches, we were able to identify that *Pseudomonas* has a key group in the activity of the core microbiome of these disparately located disease-suppressive soils. However, other taxa are mostly likely also involved, given the fact that soil suppressiveness is often governed by the combined activities of microbial consortia [51]. In our study, we also observed heightened levels of the Myxococcales and Xanthomonadales in disease-suppressive soils, and members of these groups merit further investigation for their potential roles in the control of Foc4. Also, we focused primarily on strains that showed antagonistic activities in monoculture, and it is known that many antagonistic activities are the product of interactions of strains often showing no direct individual effects [52].

**Figure 7:**
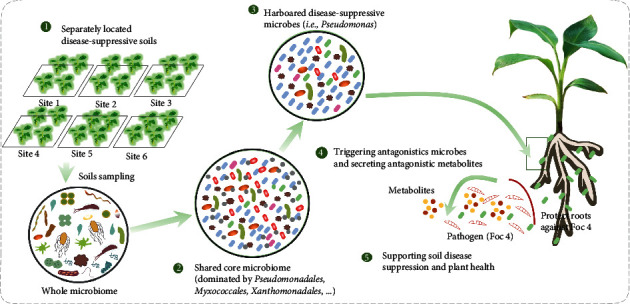
Conceptual model illustrating the shared core microbiome and potential mechanism in separately located disease-suppressive soils. Picture summarizing the features of core microbiome in separately located disease-suppressive soils and potential mechanism of disease-suppressive microbes against fungal pathogen growth and subsequent plant infection.

In conclusion, by examining six separate field locations, each with paired orchards either suppressive or conducive to *Fusarium* wilt, we were able to identify consistent microbiome signatures related to disease suppressiveness (Figure 7). Disease-suppressive soils exhibited a higher relative abundance of Myxococcales, Pseudomonadales, and Xanthomonadales. Metagenomic analysis of one pair of adjacent orchards also showed that the disease-suppressive soil harbored a higher proportion of genes related to reduced fungal virulence, quorum sensing, biofilm formation, and production of antimicrobial compounds. *Pseudomonas* was identified as a potential key taxon in disease suppression. *Pseudomonas* had a higher relative abundance in suppressive soils, and *Pseudomonas* isolates recovered from disease-suppressive fields showed a higher proportion of antagonistic activity against the pathogen as compared to those recovered from conducive soils. Despite differences in location, edaphic factors, and other microbial components of the soil community across banana cropping sites examined, we were able to detect common microbiome features related to disease suppression.

## 4. Methods

### 4.1. Field Production Survey and Basic Information of the Selected Orchards

In August 2014, a field production survey of large-scale orchards (>10 ha) was performed to detect soils suppressive to *Fusarium* wilt among the main banana plantations on Hainan Island, an important banana production area in China, where topical climatic conditions dictate spring banana harvests. To minimize the effects of cultivar, cropping year, growing period, and microclimate at each site, only banana orchards suppressive and adjacent ones conducive to *Fusarium* wilt that had been planted during the same year with the same susceptible cultivar (*Musa acuminata* Cavendish cv. Brazil) were selected for this study. Typically, a maximum disease incidence of less than 15% can be tolerated by growers when considering both the economic loss and acceptability for a subsequent crop. Therefore, orchards maintaining a disease incidence lower than 15% over long-term monoculture could be considered potentially suppressive to *Fusarium* wilt. Colocated orchards with serious *Fusarium* wilt incidences were considered as disease conducive soils. At the time of the study, six pair-located orchards monocropped over 10 years and potentially suppressive (disease incidence < 15%) or conducive (disease incidence > 50%) to *Fusarium* wilt at harvest were identified in the main banana production areas on Hainan Island.

Paired potentially suppressive and colocated conducive orchards from sites 1 and 2 were located in the southwest of Hainan Island, where typical tropical monsoon climate conditions prevail with an annual temperature (AT) of about 24°C and annual precipitation (AP) of 1150 mm on average. Orchards from sites 3 to 6 were located in the northwest of Hainan Island that experience a mean AT of 23°C and AP of 2250 mm. The planting density for orchards located in southwestern Hainan Island was approximately 2,550 plants per ha and approximately 1,950 plants per ha for orchards in the northwestern region. For colocated orchards suppressive or conducive to *Fusarium* wilt, the pesticide managements and irrigations were roughly similar based on the farm records. Worth to mention, disease-suppressive orchards were usually amended with more organic fertilizer. Detailed information about these six pair-located orchards is provided in Table S1.

### 4.2. Assay of Fusarium Wilt Incidence and Collection of Soil Samples

*Fusarium* wilt incidence was monitored according to observation of typical wilt symptoms [53]. Three representative subplots (50 m × 40 m, long × width) within each field were randomly divided for soil samples collection and disease incidence estimation in August, 2014, at the banana harvest stage. *Fusarium* wilt incidence was calculated as the proportion of infected plants among the total number of bananas planted. In each subplot, five banana trees without wilt symptoms were chosen for soil sampling according to the previously described method [54]. In total, eighteen soils from disease-suppressive orchard and eighteen soils from colocated orchards were sampled for further analysis. After removing the plant residues in soil, half of each soil sample was air-dried for chemical property measurements, and the remainder was mixed with glycerin and stored at -80°C for subsequent microbial analysis and isolation of bacterial strains.

### 4.3. Determination of Soil Chemical Properties and Extraction of Soil Genomic DNA

Soil chemical properties including soil pH, content of available phosphorus (AP), available potassium (AK), total carbon (TOC), total nitrogen (TON), ammonium nitrogen (NH_4_^+^-N), nitrate nitrogen (NO_3_^−^-N), electrical conductivity (EC), and total carbon-nitrogen ratio (C/N) were determined according to the previously described method [55]. Soil genomic DNA was extracted by using the DNeasy® PowerSoil® Kit (QIAGEN GmbH, Germany), following the manufacturer's instructions.

### 4.4. Quantification of Bacteria, Fungi, and Foc4 Abundance

Abundances of bacteria, fungi, and Foc4 were measured using a 7500 Real Time PCR System (Applied Biosystems, USA) following the established protocols with the primers of Eub338F/Eub518R for bacteria, ITS1f/5.8 s for fungi, and *FocSc-1*/*FocSc-2* for Foc4, respectively, [56, 57]. Tenfold serial dilutions of plasmids containing a full-length copy of the 16S rRNA gene from *Escherichia coli*, the 18S rRNA gene from *Saccharomyces cerevisiae*, and a fragment copy of the internal transcribed spacer (ITS) from Foc4, respectively, were used to generate standard curves. The Foc4 strain, with which the pathogenicity to banana was tested [58], was provided by our own lab. Quantitative PCR amplification for standard and DNA samples was performed in 8-well tubes with a 20 *μ*l mixture for each reaction using SYBR®Premix Ex TaqTM (TaKaRa, Japan). Each PCR reaction contained 2 *μ*l target DNA, 10 *μ*l SYBR Green Premix Ex Taq (2 ×), 0.4 *μ*l of each primer, 0.4 *μ*l ROX Reference Dye II, and sterilized water. Thermal cycling conditions for each sample were conducted according to a standard procedure with three replicates, and the results were expressed as log copy numbers g^−1^ dry soil.

### 4.5. Construction and Sequencing of Amplicon Sequencing Library

Bacterial and fungal sequencing libraries were built following the previously established protocols [59, 60]. The V4 region of bacterial 16S rRNA genes and the internal transcribed spacer 1 region (ITS1) were amplified using the primers of 515F/806R and ITS1F/ITS2, respectively. Amplicon qualities and concentrations were assessed by an Agilent 2100 Bioanalyzer Instrument (Agilent Technologies Co. Ltd, USA) and a KAPA Library Quantification Kit (KapaBiosystems, USA). Illumina HiSeq 2000 was used to sequence all constructed libraries at the Novogene Bioinformatics Institute (Beijing, China).

### 4.6. Processing of DNA Sequence Data

Raw DNA sequences with about 300 bp were split to each sample based on the unique barcodes and trimmed of the adaptor and primer sequences in QIIME (v. 1.2.0) and USEARCH (v. 9.1.13) [61]. OTU clustering was performed based on 97% pairwise identity with filtering the chimeras using the UPARSE algorithm after quality control and removal of archaea sequences [62]. The mitochondrial and nonbacterial OTUs together with OTUs whose relative abundance was lower than 0.01% were further removed. The affiliation of representative sequence for each OTU was classified against the RDP Bacterial 16S database or the UNITE Fungal ITS database using the RDP classifier (RDP, version 11.5) [63]. OTUs with occurrence frequencies higher than 80% in all conducive or suppressive soils were selected as “core OTUs” according to a previously describe method [64]. Core bacterial and fungal OTUs in conducive or suppressive soils were identified and pooled together for subsequent analysis.

The final OTU table for whole and core microbiomes was normalized using the cumulative sum scaling method [65]. Nonmetric multidimensional scaling ordination analysis was performed using the weighted UniFrac distances and was plotted using “scatterplot3d” (v. 0.3-12) in *R* 3.1.2. The significant differences of bacterial or fungal composition between disease suppressive and conducive soils were tested using the code of “adonis” in the *R* 3.1.2 “vegan” package (v. 2.6-2). Venn diagrams were then plotted to dissect microbial community composition in disease-suppressive and -conducive soils based on the all and core OTU datasets.

### 4.7. Taxonomic Analysis of the Core Microbiome

To compare the differences in taxonomic composition and to assess whether some bacterial taxa were differentially abundant in the core microbiome across samples, a three-step analysis was conducted in which the read counts were assessed separately at the phylum, order, and genus levels according to the previously described method [66]. Log2 transformed relative changes of affiliated phyla or orders in disease-suppressive soils relative to conducive soils were calculated with FDR adjusted *p* value to compare the differences in the composition of bacterial community across disease suppressive and conducive soils and plotted using the *R* 3.1.2 “ggplot2” package (v. 2.2.0). The predictors of key genera within significantly enriched orders for explaining the abundance of Foc4 in disease-conducive and -suppressive soils were identified by random forest regression analysis [67].

### 4.8. Quantification of Pseudomonas Abundance

Abundances of *Pseudomonas* were determined with the primers *Pse*435F/*Pse*686R following an established protocol by a 7500 Real Time PCR System [68]. Tenfold serial dilutions of plasmids containing a full-length copy of the 16S rRNA gene from *P. putida* were used to generate standard curves. Quantitative PCR amplification for standard and DNA samples was performed in 8-well tubes with a 20 *μ*l mixture for each reaction using SYBR®Premix Ex TaqTM (TaKaRa, Japan). Each PCR reaction contained 2 *μ*l target DNA, 10 *μ*l SYBR Green Premix Ex Taq (2 ×), 0.4 *μ*l of each primer, 0.4 *μ*l ROX Reference Dye II, and sterilized water. Thermal cycling conditions for each sample were conducted according to a standard procedure with three replicates, and the results were expressed as log copy numbers g^−1^ dry soil.

### 4.9. Greenhouse Assay of Soil Suppressiveness to Banana Fusarium Wilt

Subplots from pair-located orchards at site 3 were randomly selected at harvest to validate the soil suppressiveness phenomenon. Topsoil to a depth of 30 cm from the conducive and suppressive orchards was collected in October, 2014. This soil was used in a pot experiment to investigate the activity of disease-suppressive soil against Foc4 invasion. Four treatments with four replicates each were designed: (1) C, conducive soil without Foc4 inoculation; (2) CF, conducive soil inoculated with Foc4; (3) S, suppressive soil without Foc4 inoculation; and (4) SF, suppressive soil inoculated with Foc4. A total of eighteen pots, resulting in five or four pots each replicates, were set up for each treatment, and each pot was loaded with 4.5 kg dry soil and sown with one tissue-cultured banana seedling (*M. acuminate* Cavendish cv. Brazil). All pots were randomly placed and managed under the same conditions in the greenhouse. Two months later, pathogenic conidia of Foc4 in sterile water were added to the CF and SF treatments to give a final conidial density of 2 × 10^4^ dry weight in soil while the same volume of sterile water was irrigated into the *C* and *S* treatments. To prepare conidia, an 8 mm plug from the leading edge of a 7-day-old culture of Foc4 on potato dextrose agar medium (PDA) was placed onto the center of a fresh PDA plate , then cultured at 28 °C for 7 days, after which 5 ml sterile water wass added and through four layers gauze to harvest spores. The number of spores was estimated by a hemocytometer. Then, the same number of pathogen spores was added again to the treatments one month later. After the harvest of the first season, the second season was planted immediately, and the operation was the same as that of the first season; we here use the disease incidence of the second season.

Meanwhile, four treatments with three replicates each were designed for another greenhouse experiment as follows: (1) *C*, soil from the orchard conducive to *Fusarium* wilt; (2) *S*, soil from the orchard suppressive to *Fusarium* wilt; (3) CS, soil from the orchard conducive to *Fusarium* wilt mixed with disease-suppressive soil at a ratio of 9 : 1 (v/v); and (4) S50, soil from the orchard suppressive to *Fusarium* wilt was heated at 50°C in an oven for 2 h. Each replicate comprised ten pots while each pot was loaded with 6 kg soil and sown with one tissue-cultured banana seedling. All pots were randomly placed and managed under the same condition in the greenhouse. Symptoms of *Fusarium* wilt were monitored weekly after transplantation, and disease incidence was calculated as described above.

### 4.10. Metagenomic Sequencing of Disease-Suppressive Soil and Microbial Function Analysis

Soil from the banana orchard at site 3 and the colocated conducive orchard were selected for further metagenomic sequencing in August 2015 at the harvest stage. Five representative subplots of 50 m × 40 m from each orchard were sampled. Within each subplot, fifteen soil cores under the trunk base from five separate banana trees without wilt symptoms were sampled and pooled as a composite sample. Genomic DNA of 5 g soil from each replicate was extracted with the PowerMax®Soil DNA Isolation kit (MoBio Laboratories Inc., USA) and prepared for sequencing as described in the Illumina Paired-End Prep kit protocol. The extracted DNA was fragmented to a mean size of about 300 bp using Covaris M220 (Gene Company Limited, China), and paired-end libraries were then constructed with TruSeqTM DNA Sample Prep Kits (Illumina, San Diego, CA, USA). Adapters containing the full complement of sequencing primer hybridization sites were ligated to the blunt-ends of all fragments. Shotgun sequencing was performed on an Illumina HiSeq 4000 platform (Illumina Inc., San Diego, CA, USA) at Majorbio Bio-Pharm Technology Co., Ltd. (Shanghai, China).

The SeqPrep software (https://github.com/jstjohn/SeqPrep) was firstly used to remove the adapter sequences, and the library sickle (https://github.com/najoshi/sickle) was then used to trim the reads. If the mean quality of bases inside a window dropped below 20, the remainder of the read below the quality threshold was trimmed. Quality-trimmer reads shorter than 50 bp or containing ambiguous bases were also discarded. The filtered sequences were de novo assembled using SOAP software (http://soap.genomics.org.cn, V. 1.06). The *k*-mer value of the main splicing parameter was set in the range of 39-47. Prediction of genes of the assembled contigs to open reading frames (ORFs) was performed using MetaGene software. All of the predicted gene sequences were clustered using CD-HIT software (v. 4.6.4). Further, the longest gene was used as a representative sequence for each cluster to construct a nonredundant gene set. The high-quality reads of each sample were matched to the nonredundant gene set (95% identity) using SOAPaligner software to build a comprehensive metagenome reference gene set for further analysis. The composition difference of functions was compared based on this final gene set by conducting NMDS ordinations in the *R* 3.1.2 “vegan” package (v. 2.6-2). First, the final metagenome reference gene set was blasted using BLASTP against the NCBI nr database (June 2020) to obtain the taxonomic assignment. Then, the annotation of the gene set was blasted using BLASTP against the eggnog database to obtain Clusters of Orthologous Groups (COG) (2003 COGs, 2014 update), and against the Kyoto Encyclopedia of Genes and Genomes (KEGG) database (Release 95.2) to obtain the pathway information for genes. The annotation of the gene set was blasted using BLASTP against pathogen-host interaction (PHI) databases (v. 4.10) to obtain the functions related to pathogen and host interaction. Furthermore, the contigs with length larger than 5 kb were processed with antiSMASH (V. 5.0) with default parameters to analyze the secondary metabolism ability according to the previously described method [69].

### 4.11. Determinations of Total Culturable Bacterial Counts, Recovery of Pseudomonas Isolates, and In Vitro Tests of Antagonistic Activity against Foc4

Considering *Pseudomonas* was identified as a key specie in maintaining soil suppressiveness based on high-throughput sequencing analysis, culturable *Pseudomonas* was isolated. Five grams of the collected soil from six pair-located disease-suppressive and -conducive orchards which was mixed with glycerin and stored at -80°C previously was added to a 150 mL Erlenmeyer flask containing 45 mL of sterilized distilled water. After shaken for 30 min at 170 rpm at 30°C, 10-fold serial dilutions were made, and appropriate suspensions were loaded onto Tryptic Soy Broth with one-tenth-strength (^1^/_10_ TSB) to determine total culturable heterotrophic bacterial counts. Similarly, the same dilution series was spread onto King's medium B (KB) to enumerate culturable *Pseudomonas* and recover *Pseudomonas* isolates. All culture plates were incubated for 36 h at 30°C. Forty colonies from the KB plates for each soil sample were randomly picked to isolate potential antagonists against the Foc4 on the PDA medium using the dual culture method [70]. Isolates showing the antagonistic zones were further cultured in KB broth, and genomic DNA was then extracted using a rapid one-tube genomic DNA extraction protocol [71]. The bacterial 16S rRNA genes were amplified using the primer 27F/1492R to generate phylogenetic information.

### 4.12. Evaluating the Biocontrol Activity of Isolated Pseudomonas Strains

The effects of four *Pseudomonas* isolates showing the strongest antagonistic activity in disease-suppressive soils against banana *Fusarium* wilt were further assessed in the greenhouse of WanZhong Agricultural Company from March to May, 2016. The topsoil from conducive orchards to *Fusarium* wilt at site 3 was collected for this biocontrol test. Five treatments with three replicates each were established including conducive soil amended with 50 mL fermentation culture of the *Pseudomonas* strains S9 (T1), S20 (T2), S27 (T3), S40 (T4), and equally mixed fermentation culture from all above four strains (T5). Monocropped soil amended with 50 mL sterile KB broth (CK) was included as a control. Each replicate contained ten pots, transplanted with a single tissue-cultured banana seedling. The fermentation liquor of the various strains was added into soil near the pseudostem at weekly intervals beginning 15 days after the seedlings had been transplanted. After three months, *Fusarium* wilt was monitored weekly and measured as above.

### 4.13. Determinations of the Abundance of Pseudomonas and F. oxysporum in the Soil Amended with Isolated Pseudomonas Strains

Rhizosphere soil was sampled in May, 2016 from three banana plants without obvious infection symptoms. All roots from the replicates were pooled, then shaken gently by hand to remove the loosely adhered soil, and minced into fragments approximately 5 cm long. For each sample, about 200 g of roots were placed into a 500 mL Erlenmeyer flask previously loaded with 200 mL of sterilize distilled water. After shaken at 170 rpm for 30 min at ambient temperature, the roots were removed, and the soil slurry were then centrifuged at 4000 × g for 5 min. The plant residue was carefully removed by sterilized tweezers, genomic DNA in the sediment soil was extracted, and then the abundance of *F*. *oxysporum* and *Pseudomonas* was determined.

## Data Availability

Raw amplicon sequencing data was deposited at the National Center for Biotechnology Information (NCBI) under the accession number of PRJNA627608. All shotgun raw sequences were stored at NCBI with the accession number of PRJNA630300. The 16S rRNA gene sequences of four *Pseudomonas* isolates were deposited in the GenBank database under accession numbers MT364444-MT364447.

## Supplementary Materials

Supplementary Materials Figure S1: violin plot showing abundance of total bacteria and fungi and ratio of bacteria to fungi (*B*/*F*) in disease conducive (*C*) and suppressive (*S*) soils determined by qPCR. The *p* values in the plot were calculated based on Wilcoxon test and corrected by FDR. Figure S2: the rarefaction curves for whole bacterial and fungal communities in disease conducive (*C*) and suppressive (*S*) soils. C1-18 with pink color represent subsamples for conducive soil collected from site 1 to site 6 while S1-18 with cyan color represent subsamples for suppressive soil collected from site 1 to site 6. Figure S3: violin plot showing Shannon index of core bacterial and fungal communities in disease-conducive (*C*) and -suppressive (*S*) soils. Figure S4: stacked bar chart depicting the distribution of dominant phyla in core bacterial and fungal communities. C1-18 represent subsamples for conducive soil collected from site 1 to site 6 while S1-18 represent subsamples for suppressive soil collected from site 1 to site 6. Figure S5: violin plot showing relative abundance of five key genera which were significantly enriched in disease-suppressive soils among Myxococcales, Pseudomonadales, and Xanthomonadales. The *p* values in the plot were calculated based on Wilcoxon test and corrected by FDR. Table S1: field site information and soil chemical properties of sampled soils. *C* and *S* indicate that the soil samples were collected from orchards conducive or suppressive to banana *Fusarium* wilt, respectively. Symbol ^∗^ presents a significant difference for mean value of each soil chemical property observed between all conducive and suppressive soils based on corrected Wilcoxon test. TON represents content of soil total nitrogen, TOC represents content of soil total carbon, NH_4_^+^-N indicates content of soil ammonium nitrogen, NO_3_^−^-N represents content of soil nitrate nitrogen, EC represents soil electrical conductivity, AP represents content of soil available phosphorus, AK represents content of soil available potassium, and *C*/*N* represents ratio of soil total carbon to total nitrogen. Table S2: general information of sequencing data used for final analysis after basic quality control for whole and core microbiomes. Table S3: PERMANOVA results for whole and core bacterial and fungal community structures at the OTU level. *p* values were calculated based on 999 permutations (lowest *p* value possible is 0.001). Site means location of sampled soils, and suppression ability indicates the soils conducive or suppressive to banana *Fusarium* wilt. Table S4: relative abundance of phylum for core bacterial and fungal community in disease-conducive (*C*) and -suppressive (*S*) soils. Table S5: relative abundance of dominate orders within Proteobacteria for the core bacterial community in disease-conducive (*C*) and -suppressive (*S*) soils. Table S6: overview of sequences of shotgun-metagenome datasets representing each soil sample. Table S7: overview of genes catalog assembly to open reading frames (ORFs) (*Supplementary Materials*).
