# Supplementary material for: Shared Core Microbiome and Functionality of Key Taxa Suppressive to Banana Fusarium Wilt
Source: Research (Wash D C). 2022 Sep 15;2022:9818073. doi: 10.34133/2022/9818073 (PMC9513836; doi:10.34133/2022/9818073)
Supplement: Supplementary Materials — Figure S1: violin plot showing abundance of total bacteria and fungi and ratio of bacteria to fungi (B/F) in disease conducive (C) and suppressive (S) soils determined by qPCR. The p values in the plot were calculated based on Wilcoxon test and corrected by FDR. Figure S2: the rarefaction curves for whole bacterial and fungal communities in disease conducive (C) and suppressive (S) soils. C1-18 with pink color represent subsamples for conducive soil collected from site 1 to site 6 while S1-18 with cyan color represent subsamples for suppressive soil collected from site 1 to site 6. Figure S3: violin plot showing Shannon index of core bacterial and fungal communities in disease-conducive (C) and -suppressive (S) soils. Figure S4: stacked bar chart depicting the distribution of dominant phyla in core bacterial and fungal communities. C1-18 represent subsamples for conducive soil collected from site 1 to site 6 while S1-18 represent subsamples for suppressive soil collected from site 1 to site 6. Figure S5: violin plot showing relative abundance of five key genera which were significantly enriched in disease-suppressive soils among Myxococcales, Pseudomonadales, and Xanthomonadales. The p values in the plot were calculated based on Wilcoxon test and corrected by FDR. Table S1: field site information and soil chemical properties of sampled soils. C and S indicate that the soil samples were collected from orchards conducive or suppressive to banana Fusarium wilt, respectively. Symbol ∗ presents a significant difference for mean value of each soil chemical property observed between all conducive and suppressive soils based on corrected Wilcoxon test. TON represents content of soil total nitrogen, TOC represents content of soil total carbon, NH4+-N indicates content of soil ammonium nitrogen, NO3−-N represents content of soil nitrate nitrogen, EC represents soil electrical conductivity, AP represents content of soil available phosphorus, AK represents content of soil avail [file 9818073.f1.docx]

Supporting Online Material for

**Shared core microbiome and functionality of key taxa suppressive to banana *Fusarium* wilt**

Zongzhuan Shen^1, 2^, Linda S. Thomashow^3^, Yannan Ou^1^, Chengyuan Tao^1^, Jiabao Wang^1, 2^, Wu Xiong^1^, Hongjun Liu^1^, Rong Li^1, 2^, Qirong Shen^1, 2🖂^, George A. Kowalchuk^4^

**Author affiliation**

^1^ Jiangsu Provincial Key Lab of Solid Organic Waste Utilization, Jiangsu Collaborative Innovation Center of Solid Organic Wastes, Educational Ministry Engineering Center of Resource-saving fertilizers, The Key Laboratory of Plant Immunity, Joint International Research Laboratory of Soil Health, Nanjing Agricultural University, Nanjing, 210095, Jiangsu, People’s Republic of China

^2^ The Sanya Institute of the Nanjing Agricultural University, Sanya, Hainan Province, China

^3^ US Department of Agriculture, Agricultural Research Service, Wheat Health, Genetics and Quality Research Unit, Pullman, WA, USA

^4^ Ecology and Biodiversity Group, Institute of Environmental Biology, Department of Biology, Utrecht University, 3584 CH Utrecht, Netherlands

^🖂^Corresponding author: Qirong Shen, College of Resources and Environmental Sciences, Nanjing Agricultural University, 210095, Nanjing, China. E-mail: shenqirong@njau.edu.cn. Tel: (86)02584396291.

**Additional file**

**This additional file includes:**

Figure S1 to S5

Table S1 to S7

**Supplemental figures**

**Figure S1** Violin plot showing abundance of total bacteria and fungi, and ratio of bacteria to fungi (B/F) in disease conducive (C) and suppressive (S) soils determined by qPCR. The *p* values in the plot were calculated based on Wilcoxon test and corrected by FDR.


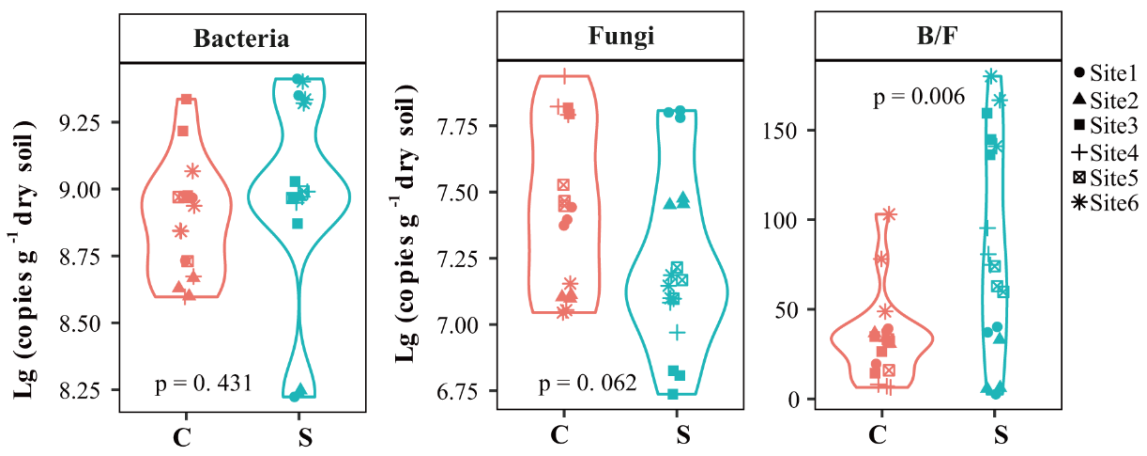


**Figure S2** The rarefaction curves for whole bacterial and fungal communities in disease conducive (C) and suppressive(S) soils. C1-18 with pink color represents subsamples for conducive soil collected from sites 1 to site 6 while S1-18 with cyan color represents subsamples for suppressive soil collected from sites 1 to site 6.


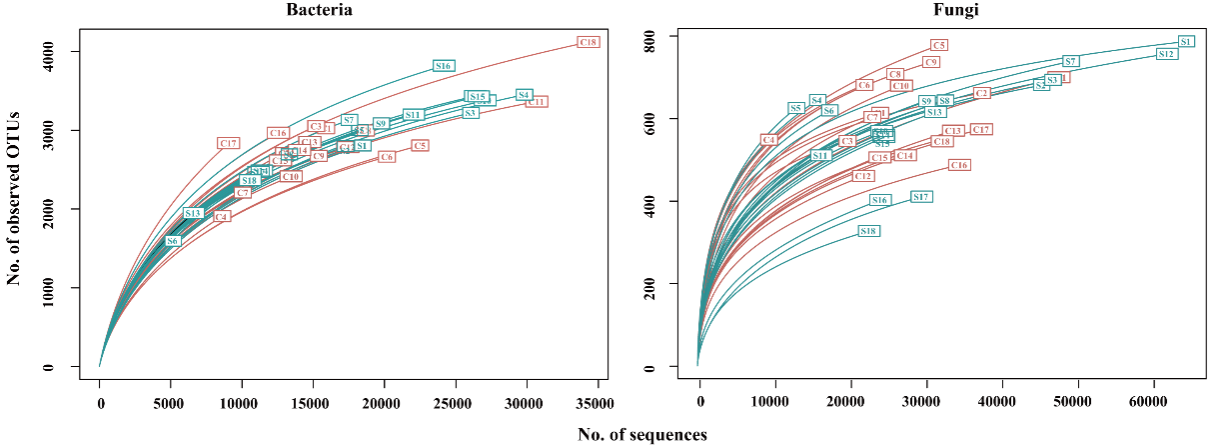


**Figure S3** Violin plot showing Shannon index of core bacterial and fungal communities in disease conducive (C) and suppressive(S) soils.


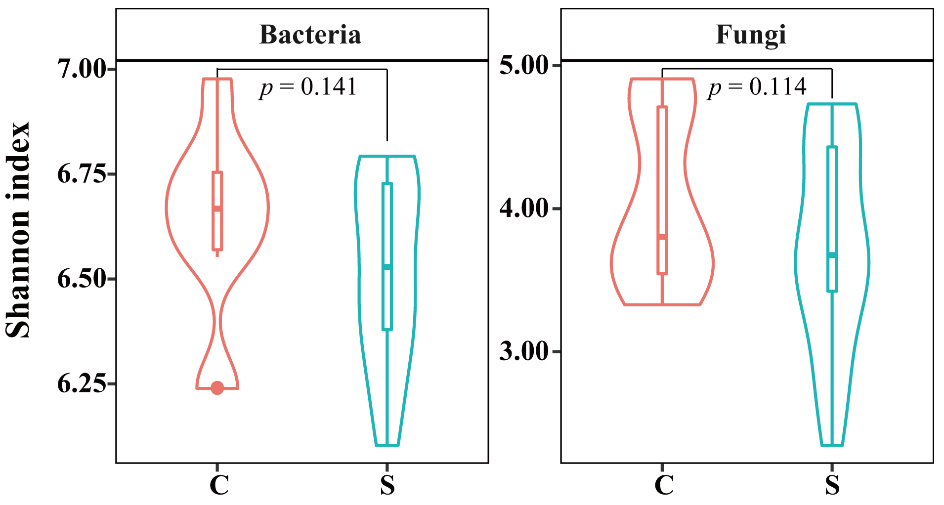


**Figure** **S4** Stacked bar chart showing dominant phyla affiliation in core bacterial and fungal communities. C1-18 represents subsamples for conducive soil collected from sites 1 to site 6 while S1-18 represents subsamples for suppressive soil collected from sites 1 to site 6.


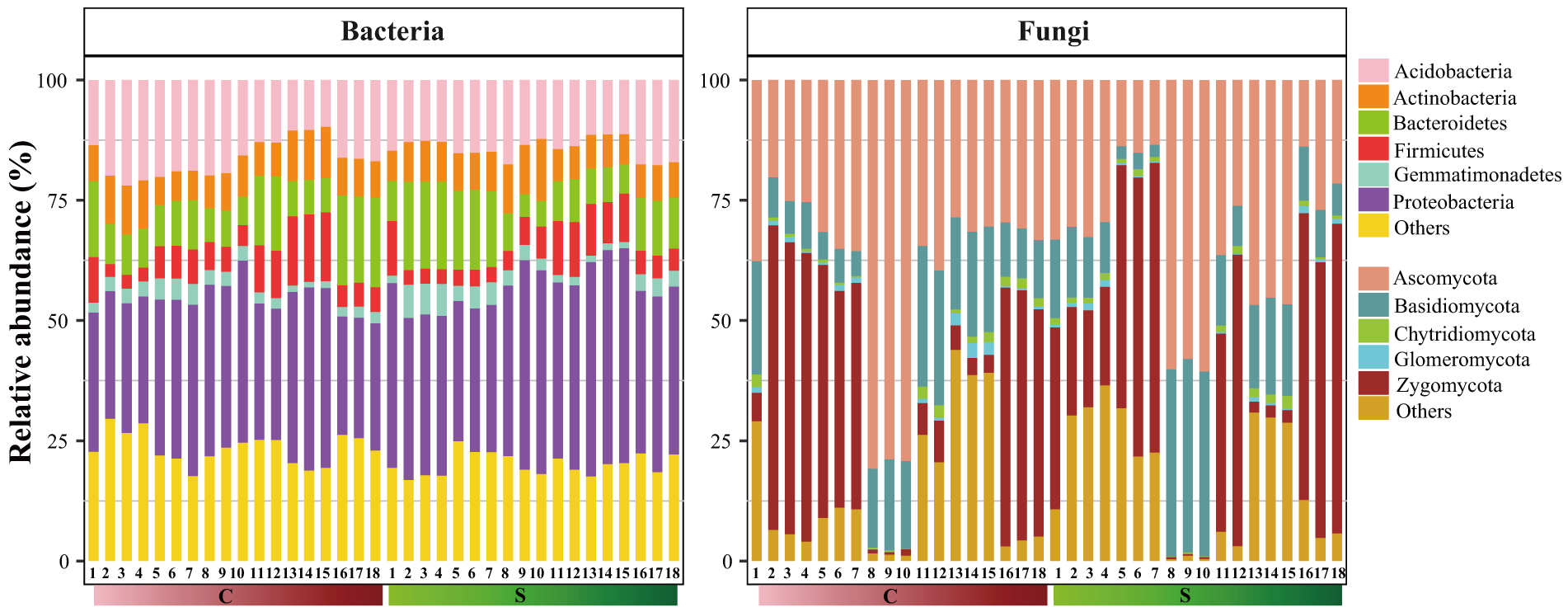


**Figure S5** Violin plot showing relative abundance of five key genera which were significantly enriched in disease suppressive soils among Myxococcales, Pseudomondales, and Xanthomondales. The *p* values in the plot were calculated based on Wilcoxon test and corrected by FDR.


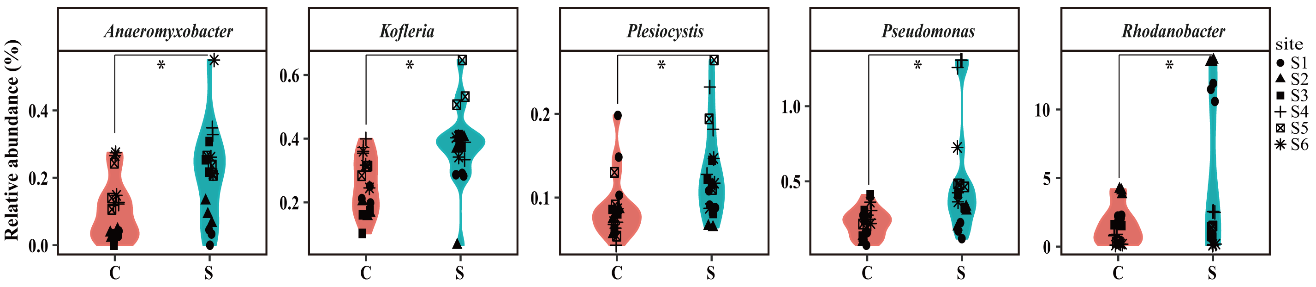


**Supplemental tables**

**Table S1** Field site information and soil chemical properties of sampled soils. C and S indicate that the soil samples were collected from orchards conducive or suppressive to banana *Fusarium* wilt, respectively. Symbol^*^ presents a significant difference for mean value of each soil chemical property observed between all conducive and suppressive soils based on corrected Wilcoxon test. TON represents content of soil total nitrogen, TOC represents content of soil total carbon, NH_4_^+^-N represents content of soil ammonium nitrogen, NO_3_^-^-N represents content of soil nitrate nitrogen, EC represents soil electrical conductivity, AP represents content of soil available phosphorus, AK represents content of soil available potassium, C/N represents ratio of soil total carbon to total nitrogen.

| **Sample**  **ID** | **Site** | **Administrative**  **division** | **Cropping**  **year** | **pH^*^** | **TON^*^**  **(g/kg)** | **TOC^*^**  **(g/kg)** | **NH_4_^+^-N**  **(mg/kg)** | **NO_3_^-^-N**  **(mg/kg)** | **EC**  **(us/cm)** | **AP^*^**  **(mg/kg)** | **AK^*^**  **(mg/kg)** | **C/N** |
| --- | --- | --- | --- | --- | --- | --- | --- | --- | --- | --- | --- | --- |
| **C1** | Site1 | Jianfeng, Ledong | 14 | 5.58±0.11 | 0.81±0.13 | 6.1 ±1.1 | 0.42±0.23 | 0.72±0.17 | 228±4 | 151± 6 | 225±8 | 7.46 ± 0.13 |
| **S1** |  |  |  | 6.04±0.19 | 1.05±0.04 | 8.2 ±0.3 | 1.47±0.19 | 2.12±0.01 | 89±5 | 192±4 | 338±8 | 7.80 ± 0.04 |
| **C2** | Site2 | Jianfeng, Ledong | 12 | 6.10±0.14 | 0.78±0.06 | 6.9±0.1 | 4.24±0.05 | 7.82±0.67 | 173±8 | 147±4 | 130±2 | 8.93 ± 0.63 |
| **S2** |  |  |  | 5.86 ±0.16 | 0.98±0.02 | 8.7±0.1 | 2.20±0.13 | 5.50 ±0.21 | 365±8 | 164±5 | 172±10 | 8.94 ± 0.22 |
| **C3** | Site3 | Fushan,Chengmai | 10 | 4.64±0.12 | 1.45±0.04 | 14.1 ±0.4 | 1.20±0.15 | 1.39±0.09 | 189±12 | 173± 4 | 251±12 | 9.67 ± 0.04 |
| **S3** |  |  |  | 6.62±0.15 | 1.70±0.05 | 15.0 ±0.2 | 0.59±0.11 | 0.79 ±0.02 | 327±9 | 231± 3 | 453±16 | 8.82 ± 0.38 |
| **C4** | Site4 | Xinying,Lingao | 12 | 4.85±0.07 | 1.52 ±0.05 | 15.1 ±0.5 | 1.74±0.04 | 4.41±0.22 | 124±4 | 155± 4 | 152± 5 | 9.94 ± 0.23 |
| **S4** |  |  |  | 6.76±0.16 | 1.75 ±0.04 | 19.4 ±0.2 | 2.31±0.12 | 1.69±0.05 | 85±5 | 184± 5 | 283±18 | 11.07 ± 0.19 |
| **C5** | Site5 | Nanbao,  Lingao | 13 | 6.00 ±0.17 | 1.75±0.06 | 16.1±0.1 | 0.65 ±0.22 | 1.06±0.07 | 117 ± 6 | 106± 8 | 258±25 | 9.24 ± 0.23 |
| **S5** |  |  |  | 6.71 ±0.22 | 1.61±0.06 | 15.4±0.4 | 0.63±0.16 | 2.28±0.07 | 164±7 | 275 ±4 | 352±10 | 9.58 ± 0.10 |
| **C6** | Site6 | Dacheng,danzhou | 16 | 6.09±0.12 | 1.25±0.04 | 14.4 ±0.1 | 1.20±0.15 | 2.17±0.08 | 160 ±5 | 173 ±5 | 303±15 | 11.50 ±0.28 |
| **S6** |  |  |  | 6.18±0.18 | 1.46±0.01 | 16.4±0.3 | 0.73±0.22 | 1.28±0.06 | 221±4 | 224±10 | 442 ±13 | 11.26 ±0.1 |
| **C (mean)** |  |  |  | 5.54 ±0.62 | 1.26±0.37 | 12.1± 4.2 | 1.57 ± 1.31 | 2.93 ± 2.58 | 165± 40 | 151 ± 24 | 220 ± 63 | 9.46 ± 1.27 |
| **S (mean)** |  |  |  | 6.36 ±0.39 | 1.42 ±0.32 | 13.9 ± 4.2 | 1.32 ±0.75 | 2.28 ± 1.57 | 208 ±111 | 212 ± 39 | 323 ± 131 | 9.58 ± 1.29 |

**Table S2** General information of sequencing data used for final analysis after basic quality control for whole and core microbiomes.

| Sample  ID | Whole | | | | |  | Core | | | | |
| --- | --- | --- | --- | --- | --- | --- | --- | --- | --- | --- | --- |
|  | No. of OTUs | |  | No. of sequences | |  | No. of OTUs | |  | No. of sequences | |
|  | Bacteria | Fungi |  | Bacteria | Fungi |  | Bacteria | Fungi |  | Bacteria | Fungi |
| C | 8,477 | 2,594 |  | 298,482 | 511,877 |  | 863 | 77 |  | 184,620 | 189,988 |
| S | 8,327 | 2,512 |  | 331,724 | 576,193 |  | 813 | 70 |  | 208,051 | 306,778 |
| Total | 9,108 | 2,936 |  | 630,206 | 1,088,070 |  | 1,033 | 92 |  | 392,671 | 496,766 |

**Table S3** PERMANOVA results for whole and core bacterial and fungal community structures at the OTU level. *p* values were calculated based on 999 permutations (lowest *p* value possible is 0.001). Site means location of sampled soils and suppression ability indicates the soils conducive or suppressive to banana *Fusarium* wilt.

| Factors | Bacteria | | | | |  | Fungi | | | | |
| --- | --- | --- | --- | --- | --- | --- | --- | --- | --- | --- | --- |
|  | All | |  | Core | |  | All | |  | Core | |
|  | F. Model | *p* |  | F. Model | *p* |  | F. Model | *p* |  | F. Model | *p* |
| Suppression ability | 3.08 | 0.001 |  | 69.82 | 0.001 |  | 2.48 | 0.023 |  | 59.93 | 0.001 |
| Site | 6.07 | 0.001 |  | 16.68 | 0.001 |  | 15.22 | 0.001 |  | 49.05 | 0.001 |
| Suppression ability × site | 2.42 | 0.001 |  | 5.81 | 0.001 |  | 2.41 | 0.001 |  | 8.10 | 0.001 |

**Table S4** Relative abundance of phylum for core bacterial and fungal community in disease conducive (C) and suppressive (S) soils.

| **Domain** | **Phylum** | **C (mean)** | **S (mean)** | **Fold Change** | ***p*** |
| --- | --- | --- | --- | --- | --- |
| Bacteria | Acidobacteria | 20.4 | 17.2 | -0.25 | 0.020 |
|  | Actinobacteria | 8.9 | 8.3 | -0.09 | 1 |
|  | Bacteroidetes | 10.7 | 10.2 | -0.07 | 1 |
|  | Firmicutes | 7.4 | 6.7 | -0.15 | 1 |
|  | Gemmatimonadetes | 2.4 | 2.5 | 0.03 | 1 |
|  | Proteobacteria | 31.8 | 38.9 | 0.29 | 0.002 |
|  | Others | 18.4 | 16.3 | -0.17 | 0.230 |
| Fungi | Ascomycota | 53.7 | 66.7 | 0.31 | 0.104 |
|  | Basidiomycota | 11.5 | 15.5 | 0.43 | 0.567 |
|  | Chytridiomycota | 0.1 | 0.1 | 0.62 | 0.813 |
|  | Fungi_unidentified | 26.7 | 14.9 | -0.84 | 0.121 |
|  | Glomeromycota | 5.1 | 2.0 | -1.38 | 0.565 |
|  | Zygomycota | 3.0 | 0.7 | -2.07 | 0.104 |

**Table S5** Relative abundance of dominate orders within Proteobacteria for the core bacterial community in disease conducive (C)and suppressive (S) soils.

| **Order** | **C (mean)** | **S (mean)** | **Fold Change** | ***p*** |
| --- | --- | --- | --- | --- |
| Burkholderiales | 3.9 | 3.6 | -0.11 | 0.671 |
| Chromatiales | 1.2 | 0.8 | -0.62 | 0.360 |
| Myxococcales | 1.5 | 2.0 | 0.46 | 0.007 |
| Oceanospirillales | 3.0 | 4.0 | 0.42 | 0.671 |
| Pseudomonadales | 1.4 | 2.72 | 0.98 | <0.001 |
| Rhizobiales | 4.8 | 4.9 | 0.036 | 0.671 |
| Rhodocyclales | 1.7 | 2.2 | 0.39 | 0.671 |
| Rhodospirillales | 1.5 | 1.3 | -0.20 | 0.360 |
| Sphingomonadales | 2.8 | 3.0 | 0.10 | 0.671 |
| Xanthomonadales | 6.0 | 8.7 | 0.52 | 0.021 |
| Others_Proteobacteria | 4.9 | 5.6 | 0.18 | 0.360 |

**Table S6** Overview of sequences merge-ability and number of annotations made for shotgun-metagenome datasets representing each soil sample.

| Sample | Raw reads | Raw bases  (Gp) | Clean reads | Clean bases  (Gp) | Percent in raw reads  (%) | Percent in raw bases  (%) | Contigs | Contigs bases  (bp) | N50  (bp) | N90  (bp) | Max  (bp) |
| --- | --- | --- | --- | --- | --- | --- | --- | --- | --- | --- | --- |
| C1 | 144,309,092 | 20.2 | 141,666,905 | 19.6 | 98.2 | 97.1 | 197,001 | 178,067,674 | 879 | 543 | 76,538 |
| C2 | 104,167,052 | 14.6 | 102,273,748 | 14.1 | 98.2 | 97.2 | 62,494 | 51,220,742 | 756 | 532 | 39,396 |
| C3 | 134,586,466 | 18.8 | 131,968,610 | 18.2 | 98.1 | 97.0 | 75,277 | 60,950,162 | 755 | 531 | 64,166 |
| C4 | 131,462,464 | 18.4 | 129,501,454 | 17.9 | 98.5 | 97.3 | 60,550 | 50,796,689 | 772 | 532 | 77,407 |
| C5 | 114,481,946 | 16.0 | 112,544,114 | 15.5 | 98.3 | 97.0 | 111,032 | 103,784,250 | 910 | 544 | 287,318 |
| S1 | 181,882,412 | 25.4 | 177,854,853 | 24.4 | 97.8 | 96.2 | 240,271 | 219,596,490 | 892 | 544 | 60,998 |
| S2 | 122,478,972 | 17.1 | 120,006,513 | 16.5 | 98.0 | 96.5 | 98,124 | 80,890,793 | 769 | 532 | 88,716 |
| S3 | 108,447,310 | 15.1 | 104,960,181 | 14.3 | 96.8 | 94.6 | 85,991 | 73,457,231 | 822 | 537 | 62,193 |
| S4 | 126,262,180 | 17.6 | 122,577,411 | 16.8 | 97.1 | 95.0 | 89,345 | 75,792,232 | 815 | 536 | 48,036 |
| S5 | 110,981,738 | 15.5 | 107,854,310 | 14.8 | 97.2 | 95.2 | 128,405 | 112,602,183 | 862 | 542 | 67,634 |

**Table S7** Overview of genes catalog assembly to open reading frames (ORFs).

| Sample | Open reading frames  (ORFs) | Total length  (bp) | Average length  (bp) | Max  (bp) |
| --- | --- | --- | --- | --- |
| C1 | 307,834 | 161,823,993 | 526 | 10,626 |
| C2 | 91,624 | 46,330,173 | 506 | 13,319 |
| C3 | 110,230 | 55,006,798 | 499 | 12,813 |
| C4 | 90,525 | 46,318,215 | 512 | 11,231 |
| C5 | 176,565 | 93,856,680 | 532 | 11,654 |
| S1 | 376,331 | 202,235,319 | 537 | 12,481 |
| S2 | 147,398 | 74,453,922 | 505 | 10,878 |
| S3 | 130,303 | 67,084,538 | 515 | 6,010 |
| S4 | 135,197 | 69,728,834 | 516 | 6,261 |
| S5 | 198,166 | 104,878,979 | 529 | 6,336 |
